# Association of Vasomotor and Other Menopausal Symptoms with Risk of Cardiovascular Disease: A Systematic Review and Meta-Analysis

**DOI:** 10.1371/journal.pone.0157417

**Published:** 2016-06-17

**Authors:** Taulant Muka, Clare Oliver-Williams, Veronica Colpani, Setor Kunutsor, Susmita Chowdhury, Rajiv Chowdhury, Maryam Kavousi, Oscar H. Franco

**Affiliations:** 1 Department of Epidemiology, Erasmus MC, University Medical Center Rotterdam, Rotterdam, The Netherlands; 2 Department of Public Health & Primary Care, Cardiovascular Epidemiology Unit, University of Cambridge, Cambridge, CB1 8RN, United Kingdom; 3 Universidade Federal do Rio Grande do Sul, Porto Alegre, Brazil; University of Utah, UNITED STATES

## Abstract

**Importance:**

Vasomotor symptoms (hot flushes and night sweats) and other symptoms, including depression, anxiety and panic attacks, are commonly experienced by menopausal women and have been associated with an unfavourable cardiovascular risk profile.

**Objective:**

To investigate whether presence of menopausal symptoms is associated with the development of cardiovascular disease (CVD).

**Methods:**

Five electronic databases (Medline, EMBASE and Web of Science) were search until February 17th, 2015 to identify relevant studies. Observational cohort studies or randomised intervention studies were eligible for inclusion if they followed participants prospectively (at least 1 year of follow-up), and reported relevant estimates on the association of any vasomotor symptoms, or other menopausal symptoms, with risk of CVD, coronary heart disease (CHD), or stroke in perimenopausal, menopausal, or postmenopausal women. Data were extracted by two independent reviewers using a pre-designed data collection form. Separate pooled relative risks (RRs) for age and non-established cardiovascular risk factors (e.g., education, ethnicity) adjusted data and for established cardiovascular risk factors and potential mediators-adjusted data (e.g., smoking, body mass index, and hypertension) were calculated.

**Results:**

Out of 9,987 initially identified references, ten studies were selected, including 213,976 women with a total of 10,037 cardiovascular disease outcomes. The age and non-established cardiovascular risk factors adjusted RRs) [95% confidence intervals] for development of CHD, Stroke and CVD comparing women with and without any menopausal symptoms were 1.34 [1.13–1.58], 1.30 [0.99–1.70], 1.48 [1.21–1.80] respectively, and the corresponding RRs adjusted for cardiovascular risk factors and potential mediators were 1.18 [1.03–1.35], 1.08 [0.89–1.32], 1.29 [0.98–1.71]. However, these analyses were limited by potential unmeasured confounding and the small number of studies on this topic.

**Conclusion:**

Presence of vasomotor symptoms and other menopausal symptoms are generally associated with an increased risk of cardiovascular disease, which is mainly explained by cardiovascular risk factors.

## Introduction

Hot flushes (also known as hot flashes) along with night sweats are collectively known as vasomotor symptoms (VMS). These well-known symptoms of the menopause are commonly experienced by menopausal women[1]. 50.3%[2] to 82.1%[3] of menopausal women experience VMS, and the intensity and duration of these symptoms also vary[3]. In addition to VMS, menopausal women often experience a range of other symptoms including anxiety, depression, irritability, fatigue, decreased libido, insomnia, and panic attacks, among others[4–7].

Menopausal transition represents a critical period in women’s lives that marks an increase in cardiovascular disease (CVD) risk. Traditional CVD risk factors, such as reduced glucose tolerance, increased blood pressure and endothelial dysfunction [8, 9], tend to become very present during menopausal transition and post-menopausal years which can partly explain the augmentation in CVD risk among women. The natural decline in oestrogen levels during menopause has been associated with the increase in CVD risk among post-menopausal women [10]. Similarly, VMS are postulated to originate from changes in brain neurotransmitters and instability in the hypothalamic thermoregulatory center brought about by decline in oestrogen levels [11].

Oestrogen supplementation therefore has been considered as the most effective treatment for these symptoms [12]. However, it remains unclear whether presence of menopausal symptoms is associated with the development of CVD[13]. Since menopausal symptoms can vary greatly between women, they might help identify women at greater CVD risk that could benefit from effective preventative strategies.

In the present study, we aimed to synthesise all available observational evidence of cardiovascular disease risk in relation to either VMS or non-vasomotor symptoms of menopause, including depression, insomnia, and panic attacks, to determine the association between VMS and non-vasomotor symptoms of menopause with risk of cardiovascular disease.

## Methods

### Data Sources, Search Strategy and Eligibility Criteria

This review was conducted in accordance with the PRISMA[14] and MOOSE[15] guidelines (S1 and S2 Appendices). Two independent authors (TM and COW), in duplication, sought studies published before February 17, 2015 (date last searched) using Medline, EMBASE and Web of Science databases. The computer-based searches combined terms related to the exposure (eg, *hot flashes*, *night sweats*) and outcomes (eg, *cardiovascular disease*, *coronary disease*), without any language restriction. Details on the search strategy are provided in S3 Appendix. Studies were sought that had reported on associations of vasomotor symptoms (defined as hot flashes and/or night sweats), or menopausal symptoms such as depression, insomnia, or panic attacks, with vascular outcomes defined as composite CVD (combination of CHD and stroke), fatal or non-fatal coronary heart disease (CHD), or stroke. CHD events included myocardial infarction, coronary artery bypass graft, ischemic heart disease or sudden cardiac death if caused by myocardial infarction and CHD deaths. Stroke included both haemorrhagic and ischemic cerebrovascular events.

### Study Selection

Observational cohort studies or randomised intervention studies were eligible for inclusion if they followed participants prospectively (at least 1 year of follow-up), and reported relevant estimates on the association of any vasomotor symptoms, or other menopausal symptoms (defined above), with risk of composite CVD, fatal or non-fatal CHD, or stroke in perimenopausal, menopausal, or postmenopausal women. Two independent reviewers, working in pairs, screened the titles and abstracts of all initially identified studies according to the selection criteria. In case of disagreement, decision was reached through consensus or consultation with a third independent author (RC and SC). Full texts were retrieved from studies that satisfied all selection criteria.

### Data Extraction

Data were extracted by two independent authors (COW and SK). A predesigned data extraction form was used to collect relevant information. This included questions on study size; study design; baseline population; location; age at baseline; duration of follow-up; reported degree of adjustment (defined as ‘+’ when RRs were adjusted for age and non-established cardiovascular risk factors (e.g., education, income, ethnicity) and “++”further adjustment for established vascular risk factors and potential mediators (e.g., body mass index, smoking status, lipids, hypertension); type and numbers of vascular outcomes and reported RRs. Additionally, in the case of multiple publications, the most up-to-date or comprehensive information was included.

### Assessing the Risk of Bias

Bias within each individual study was evaluated by two independent reviewers (TM and COW) using the validated Newcastle-Ottawa Scale, a semi-quantitative scale designed to evaluate the quality of nonrandomized studies[16]. Study quality was judged on the selection criteria of participants, comparability of cases and controls, and exposure and outcome assessment. Studies that received a score of nine stars were judged to be of at low risk of bias; studies that scored seven or eight stars were considered at medium risk; those that scored six or less were considered at high risk of bias.

## Data Synthesis and Analysis

The inverse variance weighted method was used to combine relative risks to produce a pooled relative risk using random- effects meta-analysis models to allow for between study heterogeneity. Separate summary estimates were obtained for age and potential cardiovascular risk factors adjusted data (e.g., education, income, ethnicity) and for established risk factors and potential mediators-adjusted data (e.g., body mass index, smoking status, lipids, hypertension). Heterogeneity was assessed using the Cochrane χ2 statistic and the I2 statistic. Publication bias was evaluated through a funnel plot and Egger’s test. All tests were two-tailed and p-values of 0.05 or less were considered significant. STATA release 13 (Stata Corp, College Station, Texas) was used for all statistical analyses.

## Results

**Fig 1:**
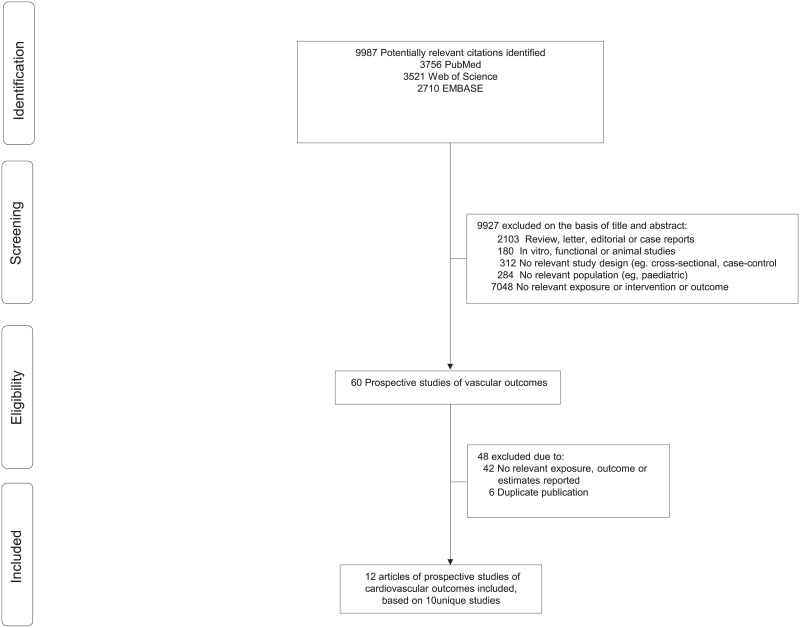
Flow diagram of studies included in the current review.

The search strategy identified 9,954 unique citations. Following initial screening based on titles and abstracts, 54 articles were retrieved and evaluated further. Of these articles, 48 were excluded for reasons shown in Fig 1 and S4 Appendix. The remaining 12 articles[17–28] (based on 10 distinct studies) were included in the review and meta-analysis (Table 1). Consequently, 213,976 individuals were included within the meta-analysis, with a total of 10,037 CVD outcomes. The average follow-up ranged from 5.3 to 15 years (Table 1). Eight studies included women from the USA, whilst the remaining study included Dutch, Swedish and Finish participants. The age of participants ranged from 30 to 89 years. Three studies assessed VMS whereas the rest of the included studies assessed other menopause symptoms; 4 studies assessed depression, 2 sleep disturbance and insomnia, and 1 study panic attacks (S1 Table). All available studies were prospective cohorts in design which were judged as medium or high-quality studies, with few potential source of bias coming from participant selection (Table 2).

**Table 1:** General characteristics of prospective studies of cardiovascular disease outcomes included in review.

Lead Author, Publication Date	Name of study	Location	Baseline survey	Baseline age range, yr	Population source	Average follow up, yr	No of total participants	No. of cases	Outcome	Covariates adjusted for
Ahto, 2007[24]	-	Finland	1991–1991	≥64	Population register	12^c^	378	21	CHD mortality	Age, marital status, social status, number of medicines.
Penninx, 1998[23]; Mendes de Leon, 1998[28]	EPESE	USA	1988	≥65	Healthcare register.	4^a^	2812	557	Incident CVD, CHD and CHD mortality	Age, sex, smoking, alcohol intake, body mass index, blood pressure, history of stroke, diabetes, cancer and physical disability.
Chandola, 2010[27]	WHII	USA	1985–1988	35–55	Healthcare register	15	3234	450	Incident CHD	No adjustment
Ferketich, 2000[25]	NHANES I	USA	1982–1984	≥30	Healthcare register	8.3	5006	187	Non-fatal CHD and CHD mortality	Age, poverty index, smoking, hypertension, diabetes and body mass index.
Gast, 2011[17]	EPOS/ WHILA	Netherlands & Sweden	1995–2000	46–64	Screening program	10.3	10,787	606	Incident CHD	Age, education, smoking, physical activity, ovariectomy, hormone therapy use, oral contraceptive use, menopausal state, BMI, SBP, DBP, total cholesterol
Sands-Lincoln, 2013[18]	WHI	USA	1993–1998	50–79	Healthcare register	10.3	86,329	7,257	CVD and incident CHD	Age, race, education, income, smoking, BMI, physical activity, alcohol intake, depression, diabetes, high BP, hyperlipidemia, comorbid conditions
Smoller, 2007[19]	MIMS	USA	1997–2000	51–83	Healthcare register	5.3	3,369	81	Incident CHD and Stroke.	Age, race, income, BMI, alcohol, hormone use, high cholesterol requiring medication, history of DM treatment, smoking, depression, history of AF, hypertension status, moderate to strenuous activity for longer than 20 minutes 3 or more times a week, history of MI, CABG, PTCA, stroke before questionnaire administration
Svartberg, 2009[21]	Rancho Bernado	USA	1984–1987	50–89	Population register	11.5	867	194	CHD mortality	Past or current estrogen and/or progestin use, BMI, exercise, smoking
Whooley, 1998[26]	SOF	USA	1988–1990	≥67	Healthcare register	6	7518	127	CHD mortality	Age, history of myocardial infarction, stroke, chronic obstructive pulmonary disease, hypertension, diabetes, smoking, perceived health and cognitive function.
W-Smoller, 2004[20]; Szmuilowicz 2011[22]	WHI-OS	USA	1994–1998	50–79	Healthcare register	9.7	93,676	2,557	CVD, incident CHD and Stroke.	Age, race, education, income, BMI, cholesterol, smoking, hormone therapy, physical activity, and hypertension status
*Total*							*213*,*976*	*12*,*037*		

^a^ median

^c^ total follow-up AF, atrial fibrillation; BMI, body mass index; CABG, coronary bypass graft surgery; CHD, coronary heart disease; CVD, cardiovascular disease; DM, diabetes mellitus; DBP, diastolic blood pressure; EPESE, Established Populations for Epidemiological Studies of the Elderly; EPOS, Eindhoven Perimenopausal Osteoporosis Study; MI, myocardial infarction; MIMS, Myocardial Ischemia and Migraine Study; PTCA, percutaneous transluminal coronary angioplasty; BP, systolic blood pressure; SOF, Study of Osteoporotic Fractures; WHILA, Women’s Health in the Lund Area; WHI-OS, Women’s Health Initiative Observational Study.

**Table 2:** Assessment of study quality through the Newcastle-Ottawa Scale.

Lead Author, Publication Date	Selection	Comparability	Outcome
W-Smoller, 2004; Szmuilowicz 2011	**	**	***
Smoller, 2007	**	**	**
Svartberg, 2009	**	*	***
Gast, 2011	***	**	***
Sands-Lincoln, 2013	***	**	***
Penninx, 1998, Mendes de Leon, 1998	***	**	**
Ahto, 2007	**	*	***
Ferketich, 2000	**	**	***
Whooley, 1998	**	*	***
Chandola 2010	**		***

Stars are awarded such that the highest quality studies with low risk of bias are awarded up to nine stars

### Association of Menopausal Symptoms and CHD

**Fig 2:**
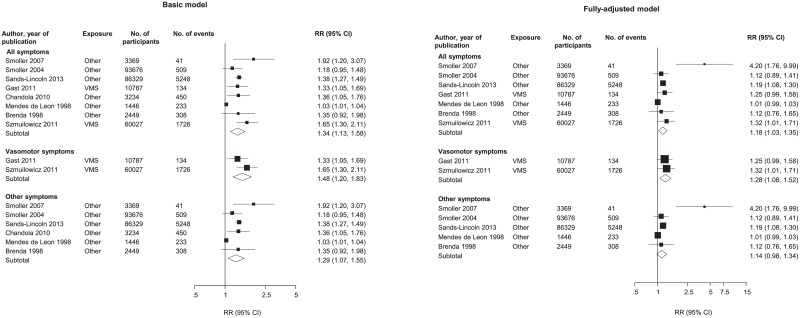
Relative risks of coronary heart disease associated with menopausal symptoms. Basic model: adjusted for age and non-established cardiovascular risk factors; Fully-adjusted model, adjusted for established cardiovascular risk factors and potential mediators. VMS, vasomotor symptoms; vasomotor symptoms include hot flashes and /or night sweats; All, includes vasomotor and other menopausal symptoms; Other, includes menopausal symptoms such as depression, insomnia, and panic attacks. Assessment of heterogeneity for basic model: All symptoms, X2 7 = 81.22, I2 = 91%, 85 to 95%; P < 0.001; vasomotor symptoms, X2 1 = 1.55, I2 = 35%; P = 0.213; other symptoms, X2 5 = 63.48, I2 = 92%, 86 to 96%; P < 0.001. Assessment of heterogeneity for fully-adjusted model: All symptoms, X2 6 = 29.20, I2 = 79%, 58 to 90%; P < 0.001; vasomotor symptoms, X2 1 = 0.09, I2 = 0%; P = 0.762; other symptoms, X2 4 = 22.63, I2 = 82%, 59 to 92%; P < 0.001.

Menopausal symptoms, either VMS or other symptoms, in relation to risk of developing CHD were reported in ten prospective observational studies, three of which assessed VMS and seven assessed other menopausal symptoms. Pooled RRs for subsequent development of CHD, adjusted for age along with other potential cardiovascular risk factors, were 1.34 (95%CI: 1.13–1.58) when comparing women with and without VMS (70,814 participants, 1,860 events), and 1.48 (95% CI: 1.20–1.83) when comparing women with and without non-VMS (190,503 participants, 6,789 events) (Fig 2). Whilst the pooled RR, adjusted for age and other potential cardiovascular risk factors, comparing women with and without any menopausal symptoms, using data from 261,317 women with 8,649 CHD outcomes, was 1.34 (95%CI: 1.13–1.58) (Fig 2). Further adjustment for established cardiovascular risk factors and potential mediators attenuated but did not abolish the associations of VMS (RR = 1.28; 95%CI = 1.08; 1.52) and of any menopausal symptoms (RR = 1.18; 95%CI = 1.03; 1.35) with CHD. In contrast, there was no significant association between non-VMS and the risk of CHD after adjustment for established cardiovascular risk factors and potential mediators (RR = 1.14; 95%CI = 0.98; 1.34) (Fig 2). There was evidence of between-study heterogeneity for non-VMS and any for menopausal symptom analyses (I^2^ = 92% (95%CI: 86%, 96%) and χ^2^: p<0.001 for non-vasomotor symptoms and I^2^ = 91% (95%CI:85%, 95%) and χ^2^: p<0.001 for all menopausal symptoms). There was no evidence of heterogeneity for the VMS analysis (I^2^ = 35% and χ^2^: p = 0.21 for VMS) (Fig 2). Restricting the CHD outcome to fatal CHD events only, no associations were found between any menopausal symptoms, VMS or non-VMS, with fatal CHD in both models (S1 Fig).

### Association of Menopausal Symptoms and Stroke

**Fig 3:**
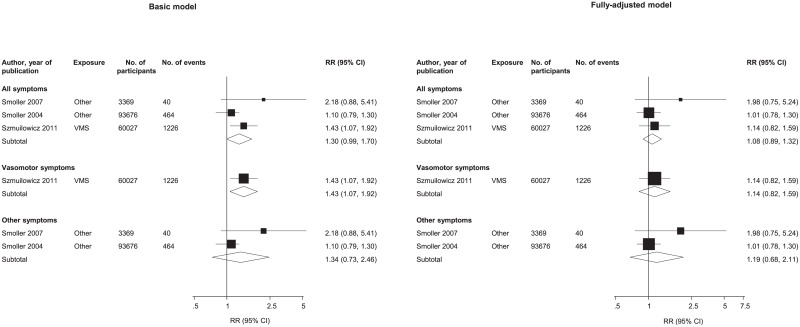
Relative risks of stroke associated with menopausal symptoms. Basic model: adjusted for age and non-established cardiovascular risk factors; Fully-adjusted model, adjusted for established cardiovascular risk factors and potential mediators. VMS, vasomotor symptoms; vasomotor symptoms include hot flashes and /or night sweats; All, includes vasomotor and other menopausal symptoms; Other, includes menopausal symptoms such as depression, insomnia, and panic attacks. Assessment of heterogeneity for basic model: All symptoms, X^2^_2_ = 3.26, I^2^ = 39%, 0 to 81%; *P* = 0.196; other symptoms, X^2^_1_ = 2.03, I^2^ = 51%, 0 to 87%; *P* = 0.154. Assessment of heterogeneity for full-adjusted model: All symptoms, X^2^_2_ = 1.86, I^2^ = 0%, 0 to 90%; *P* = 0.395; other symptoms, X^2^_1_ = 1.72, I^2^ = 42%; *P* = 0.189.

Menopausal symptoms in relation to subsequent stroke risk were reported in three prospective observational studies; VMS was evaluated by one study and two studies assessed non-VMS. From the single study evaluating stroke risk in women with and without VMS, the age and potential risk factors- adjusted RR was 1.43 (95% CI: 1.07–1.92) (60,027 participants, 1226 stroke events) (Fig 3). Age and potential cardiovascular risk factors- adjusted summary estimate of the RR was 1.34 (95%CI: 0.73–2.46) when comparing women with and without non-VMS (97,045 participants, 504 stroke events) (Fig 3). The pooled RR for subsequent stroke, adjusted for age and potential cardiovascular risk factors, was 1.30 (95% CI: 0.99–1.70) when comparing the presence and absence of any menopausal symptoms (157,072 participants, 1,727 stroke events) (Fig 3). After multiple adjustments for cardiovascular risk factors and potential mediators, the overall summery estimates of the RRs decreased to 1.14 ((95% CI: 0.82–1.59), 1.19 (95% CI: 0.68–2.11) and 1.08 (95% CI: 0.89–1.32) for VMS, non-VMS and any menopausal symptoms respectively (Fig 3). There were some evidence of between study heterogeneity, however, in either meta-analysis: I^2^ = 51% and χ^2^: p = 0.15 for non-vasomotor symptoms and I^2^ = 39% and χ^2^: p = 0.20 for all menopausal symptoms.

### Association of Menopausal Symptoms and CVD

**Fig 4:**
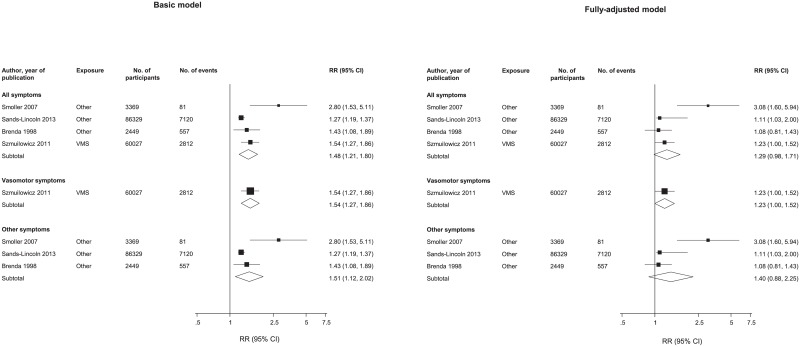
Relative risks of cardiovascular disease associated with menopausal symptoms. Basic model: adjusted for age and non-established cardiovascular risk factors; Fully-adjusted model, adjusted for established cardiovascular risk factors and potential mediators. VMS, vasomotor symptoms; vasomotor symptoms include hot flashes and /or night sweats; All, includes vasomotor and other menopausal symptoms; Other, includes menopausal symptoms such as depression, insomnia, and panic attacks. Assessment of heterogeneity for basic model: All symptoms, X2 3 = 9.95, I2 = 70%, 13 to 90%; P = 0.019; other symptoms, X2 2 = 7.05, I2 = 72%, 4 to 92%; P = 0.045. Assessment of heterogeneity for full-adjusted model: All symptoms, X2 3 = 8.68, I2 = 65%, 0 to 88%; P = 0.034; other symptoms, X2 2 = 8.67, I2 = 77%, 25 to 93%; P = 0.013.

Four cohort studies reported menopausal symptoms in relation to subsequent risk of combined CVD outcomes. All of these studies included American participants. Only one study examined the association between VMS and the risk of CVD and showed a RR adjusted for age and potential risk factors of 1.54 (1.27–1.86) (60,027 participants, 2,812 CVD events) for women experiencing VMS compared to women who did not (Fig 4). Pooled RRs for subsequent development of CVD, adjusted for age along with other potential established cardiovascular risk factors was 1.51 (95% CI: 1.12–2.02) (92,147 participants, CVD 7,758 events) when comparing women with and without non-VMS (Fig 4). The pooled RR for CVD, adjusted for age but not for conventional cardiovascular risk factors, comparing women with and without any menopausal symptoms was 1.48 (95%CI: 1.21–1.80) (152,174 participants with 10,570 CVD events, Fig 4). Multiple adjustment for established cardiovascular risk factors attenuated the overall RRs: 1.23 (95% CI: 1.00–1.52) for VMS, 1.40 (95% CI: 0.88–2.25) for non-VMS and 1.29 (95% CI: 0.98–1.71) for any menopausal symptom (Fig 4). There was evidence of between-study heterogeneity in these meta-analysis: I^2^ = 72% (95%CI: 4%, 92%) and χ^2^: p = 0.045 for non-VMS; I^2^ = 7% (95%CI: 12%, 92%) and I^2^ = 70% (95%CI: 13%, 90%) and χ^2^: p = 0.02 for all menopausal symptoms combined.

### Publication Bias

Under visual examination, Begg’s funnel plots for studies assessing the risk of CHD (S2 Fig) or combined cardiovascular outcomes (S2 Fig) were not symmetrical, and therefore, providing evidence for publication bias. This was further supported by the results of Egger’s test which were significant for both CHD and composite CVD, in particular for the established cardiovascular risk factors and potential mediators-adjusted data (S2 Fig). No evidence of publication bias was observed for fatal CHD analysis, either graphically from the funnel plot (S2 Fig) or quantitatively (P = 0.60 for Egger’s test asymmetry).

## Discussion

This is the first meta-analysis investigating the association between the presence of menopausal symptoms and subsequent development of cardiovascular disease. Overall we found that VMS and other menopausal symptoms are associated with increased risk of CHD, stroke or CVD. While the associations observed for menopausal symptoms with stroke and overall CVD could be explained, at least in part, by established cardiovascular risk factors; the association between menopausal symptoms and CHD was only partially attenuated, however, remained significant once corrected for cardiovascular risk factors and potential mediators.

Our study showed that VMS are associated with an increased risk of CVD, which was attenuated after adjustment for cardiovascular risk factors. These results are in line with the notion that menopausal symptoms may reflect an increased risk of CVD[13]. As well as other menopausal symptoms, VMS might serve as a CVD risk marker, as women with VMS may have a higher prevalence of other CVD risk factors [9, 13, 29, 30], with conventional CVD risk factors (e.g., hypertension and total cholesterol) being possible mediators in the association between VMS and CVD. Although epidemiological studies such as those included in this review cannot provide causal evidence, some previous findings indicate that menopausal women with VMS have a higher prevalence of CVD risk factors including higher blood pressure and cholesterol levels [9, 29].

On the other hand, mental health disorders, including depression, anxiety and panic attacks are already known to be CVD risk factors in both men and women of any age [31–33]. Short and long sleep duration, indicative of poor physical and mental health, has also been associated with CVD in the general population [34]. These different risk factors may work in tandem, as for instance depression has been implicated in the association between sleep quality and CVD [35]. It has also been suggested that these symptoms affect CVD risk through established risk factors such as raised BMI [36, 37]. Moreover, depression and anxiety may result in autonomic activation, leading to increased blood pressure and heart rate and decreased endothelial function [38, 39] or through affecting coagulation, by increasing platelet activity and plasma viscosity among others [38]. The sympathetic nervous activation following the panic attacks could also mediate an increased CVD risk [40].

Any results regarding the association between menopausal symptoms and CVD should be cautiously interpreted, as women with symptoms are more likely to commence hormone therapy than women without symptoms. Hormone therapy is taken to ameliorate menopausal symptoms, but might adversely affect the development of CVD [8]. Although several of the included studies adjusted the associations for hormone therapy use, the possibility of residual confounding cannot be entirely eliminated. However, the most common reason for using hormone therapy is VMS [41]; therefore if there was residual confounding by hormone therapy use, an association with VMS over and above other menopausal symptoms would be expected.

Our results might indicate the potential usefulness of menopausal symptoms in assessing CVD risk among women. As up to 20% of CHD events in women occur in the absence of conventional risk factors [42], better risk assessment among women is warranted. The magnitude of the observed association between menopausal symptoms and CVD (RR of 1.54) is similar to the estimates reported for other female-specific CVD risk factors such as gestational diabetes[43] and pregnancy-induced hypertension[44], which have recently been implicated for CVD risk classification among women by the American Heart Association[45]. Considering this potential, subsequent studies to further evaluate these associations are warranted.

This review underscores a number of gaps in the literature concerning CVD risk and menopausal symptoms. For instance (i) not all available studies assessed the severity or duration of menopausal symptoms, (ii) and interaction with the age and/or stages of menopause at which women experienced these symptoms was not consistently evaluated. Recent evidence shows that the predictive value of VMS for clinical CVD events may vary with onset of VMS at different stages of menopause; e.g. Szmuilowicz et al. found that early VSM were not associated with increased CVD risk, while late VMS were associated with increased CHD risk and all-cause mortality[46]. Also, VMS were often grouped together, impeding the assessment of individual symptoms. Experiencing menopausal symptoms in isolation or in combination might carry different cardiovascular risk[17],[21].

This review involved aggregate data from 213,613 women from the general population with over 10,000 cardiovascular events. A number of limitations, however, need to be considered. A number of studies did not adjust for several CVD risk factors; including alcohol consumption[17, 20–22] and socioeconomic status[17, 19–23]. Moreover, the results might also be subject to residual confounding. Due to using self-reported exposure and not specifying whether women with CVD at baseline were excluded, most studies failed to score well on selection as indicated by the Newcastle-Ottawa Scale. Although there is a risk of reverse-causation, one study excluded events that occurred in the first 6 months of follow-up, which had no material effect on the association[20]. Between-study heterogeneity was found in a number of analyses, which may have resulted from different levels of adjustment and differing methods of case ascertainment (ranging from death certificates to self-report validated by record linkage). Also, another potential source of bias include possible misclassification of exposure. The age-range of women included in our analysis varies across the included studies, with three studies including women age 64 and above, and therefore the symptoms can occur beyond the menopausal transition. However, two of these studies examined the association of menopausal symptoms with CHD-mortality, and therefore, may have had little or no impact on the other results reported in this systematic review. Furthermore, VMS and other menopausal symptoms were assessed by questionnaire which is subject to some measurement error. However, because the outcome in all studies included in this systematic review was assessed prospectively, the subjective measure of menopausal symptoms would likely lead to non-differential misclassification with respect to the outcome, and therefore would likely bias our estimates toward the null in our analysis. Also, a major limitation of the studies included in our systematic review is the reliance on retrospective self-report of menopausal symptoms, particularly of VMS, which are subject to fault memory and reporting bias. Assessing VMS physiologically with, for example, an ambulatory hot flash monitor to measure skin conductance, would be an alternative objective measure of vasomotor symptoms. Also, the studies varied on the time when they assessed the presence of menopausal symptoms, which may be an important factor in determining the risk for CVD. Data from the Women’s Health Initiative suggest that the predictive value of vasomotor symptoms for cardiovascular risk may vary with onset of vasomotor symptoms at different stages of menopause[22]. Furthermore, studies usually combined assessment of VMS frequency and severity, constructs which are not interchangeable. The majority of studies were based in a single country (USA). Given that CVD rates, menopausal symptoms and age at menopause[47] all vary between countries, it is possible that the strength or existence of the associations also varies by population. Therefore, to improve the generalizability of the findings, these associations would need to be evaluated in other populations. Finally, we found evidence of reporting bias in our systematic review, and therefore, some relevant studies may have been overlooked in the current systematic review.

## Conclusion

The evidence from observational studies indicate that women who experience VMS and other menopausal symptoms, including depression, anxiety and panic attacks during menopause are at greater risk of developing CVD. Further studies are needed to reliably establish which menopausal symptoms are independently associated with CVD outcomes and further clarify the potential mechanisms behind these associations.

## Supporting Information

S1 Appendix PRISMA 2009 check-list. (DOCX)

S2 Appendix MOOSE Checklist. (DOCX)

S3 Appendix Literature search strategy for prospective studies of cardiovascular outcomes. (DOCX)

S4 Appendix Forty-eight full-text excluded articles. (DOCX)

S1 Fig Relative risks of fatal CHD associated with menopausal symptoms. Basic model: adjusted for age and non-established cardiovascular risk factors; Fully-adjusted model, adjusted for established cardiovascular risk factors and potential mediators. VMS, vasomotor symptoms; vasomotor symptoms include hot flashes and /or night sweats; All, includes vasomotor and other menopausal symptoms; Other, includes menopausal symptoms such as depression, insomnia, and panic attacks.
(PDF)

S2 Fig Egger’s test in studies of menopausal symptoms and the risk cardiovascular disease. The dotted lines show 95% confidence intervals around the overall summary estimate calculated using a fixed effect model;; P-values for bias calculated using Egger’s test was 0.070 for all menopausal symptoms-CVD risk in basic adjusted analysis; 0.041 for all menopausal symptoms-CVD risk in fully- adjusted analysis; 0.016 for all menopausal symptoms-CHD risk in basic adjusted analysis; 0.083 for other menopausal symptoms-CHD risk in basic adjusted analysis; 0.014 for all menopausal symptoms-CHD risk in fully-adjusted analysis; 0.098 for other menopausal symptoms-CHD risk in fully-adjusted analysis; 0.599 for all menopausal symptoms-fatal CHD risk in basic adjusted analysis; and 0.824 for all menopausal symptoms-fatal CHD risk in fully-adjusted analysis respectively.
(PDF)

S1 Table Exposure and methods of assessment used in the studies included in the review. (DOCX)
