# Supplementary material for: Association of Vasomotor and Other Menopausal Symptoms with Risk of Cardiovascular Disease: A Systematic Review and Meta-Analysis
Source: PLoS One. 2016 Jun 17;11(6):e0157417. doi: 10.1371/journal.pone.0157417 (PMC4912069; doi:10.1371/journal.pone.0157417)
Supplement: S3 Appendix — (DOCX) [file pone.0157417.s003.docx]

Relevant studies, published before February 17, 2015 (date last searched), were identified through electronic searches not limited to the English language using MEDLINE, EMBASE, and Web of Science databases. Electronic searches were supplemented by scanning reference lists of articles identified for all relevant studies (including review articles), by hand searching of relevant journals and by correspondence with study investigators. The computer-based searches combined search terms related to markers related to vasomotor symptoms, related to vascular outcomes and related to study design without language restriction.

(hot flashes[MeSH Terms] OR “night sweat”[Text Word] OR “hot flash”[ All Fields] OR “sweat”[ All Fields] OR “flush”[ All Fields] OR “hot flush”[ All Fields] OR “ember flash”[All Fields] OR “vasomotor symptom”[All Fields] OR menopause[MeSH Terms] OR “menopause”[All Fields] OR perimenopause[MeSH Terms] OR postmenopause[MeSH Terms])

AND

(“Cardiovascular Diseases” [Mesh] OR “Coronary Artery Disease” [MeSH] OR “Atherosclerosis” [MeSH] OR “Coronary Disease” [MeSH] OR “Myocardial Infarction” [MeSh] OR “Myocardial Ischemia” [MeSH] OR “Stroke” [MeSH])

AND

(“longitudinal studies"[MeSH Terms] OR "prospective"[All Fields] OR “cohort”[All Fields] OR “follow up”[All Fields] OR ("Clinical Trials as Topic"[Mesh]) OR "Randomized Controlled Trial" [Publication Type])
