# Supplementary material for: Association of Vasomotor and Other Menopausal Symptoms with Risk of Cardiovascular Disease: A Systematic Review and Meta-Analysis
Source: PLoS One. 2016 Jun 17;11(6):e0157417. doi: 10.1371/journal.pone.0157417 (PMC4912069; doi:10.1371/journal.pone.0157417)
Supplement: S4 Appendix — (DOCX) [file pone.0157417.s004.docx]

**A. Duplicate publications**

1. Baba Y, Ishikawa S, Amagi Y, Kayaba K, Gotoh T, Kajii E. Premature menopause is associated with increased risk of cerebral infarction in Japanese women. Menopause-the Journal of the North American Menopause Society. 2010;17(3):506-10. doi: 10.1097/gme.0b013e3181c7dd41. PubMed PMID: WOS:000277501600012.

2. Gallicchio L, Miller SR, Zacur H, Flaws JA. Hot flashes and blood pressure in midlife women. Maturitas. 2010;65(1):69-74. Epub 2009/12/01. doi: S0378-5122(09)00394-6 [pii]

10.1016/j.maturitas.2009.10.013. PubMed PMID: 19945805; PubMed Central PMCID: PMC2815260.

3. Gast GC, Pop VJ, Samsioe GN, Grobbee DE, Nilsson PM, Keyzer JJ, et al. Vasomotor menopausal symptoms are associated with increased risk of coronary heart disease. Menopause. 2011;18(2):146-51. Epub 2010/12/04. doi: 10.1097/gme.0b013e3181f464fb. PubMed PMID: 21127438.

4. Pines A. Vasomotor symptoms and cardiovascular disease risk. Climacteric. 2011;14(5):535-6. doi: 10.3109/13697137.2011.599058. PubMed PMID: WOS:000294827100004.

5. Thurston RC, El Khoudary SR, Sutton-Tyrrell K, Crandall CJ, Gold EB, Sternfeld B, et al. Vasomotor Symptoms and Lipid Profiles in Women Transitioning Through Menopause. Obstet Gynecol. 2012;119(4):753-61. doi: 10.1097/AOG.0b013e31824a09ec. PubMed PMID: WOS:000301971400010.

6. Tuomikoski P, Mikkola TS, Tikkanen MJ, Ylikorkala O. Hot flushes and biochemical markers for cardiovascular disease: a randomized trial on hormone therapy. Climacteric. 2010;13(5):457-66. Epub 2010/05/07. doi: 10.3109/13697131003624656. PubMed PMID: 20443719.

**B. No relevant exposure, outcome or estimates reported**

1. Albert CM, Chae CU, Rexrode KM, Manson JE, Kawachi I. Phobic anxiety and risk of coronary heart disease and sudden cardiac death among women. Circulation. 2005;111(4):480-7. doi: 10.1161/01.Cir.0000153813.64165.5d. PubMed PMID: WOS:000226692600018.

2. Allison MA, Manson JE, Aragaki A, Langer RD, Rossouw J, Curb D, et al. Vasomotor symptoms and coronary artery calcium in postmenopausal women. Menopause-the Journal of the North American Menopause Society. 2010;17(6):1136-45. doi: 10.1097/gme.0b013e3181e664dc. PubMed PMID: WOS:000283993700011.

3. Archer DF, Pinkerton JV, Guico-Pabia CJ, Hwang E, Cheng RFJ. Cardiovascular, Cerebrovascular and Hepatic Safety of Desvenlafaxine Over 1 Year in Women With Vasomotor Symptoms Associated With Menopause. Menopause-the Journal of the North American Menopause Society. 2011;18(12):1348-. PubMed PMID: WOS:000297914800061.

4. Baba Y, Ishikawa S, Amagi Y, Kayaba K, Gotoh T, Kajii E. Premature menopause is associated with increased risk of cerebral infarction in Japanese women. Menopause. 2010;17(3):506-10. Epub 2010/01/01. doi: 10.1097/gme.0b013e3181c7dd41. PubMed PMID: 20042893.

5. Bechlioulis A, Kalantaridou SN, Naka KK, Chatzikyriakidou A, Calis KA, Makrigiannakis A, et al. Endothelial Function, But Not Carotid Intima-Media Thickness, Is Affected Early in Menopause and Is Associated with Severity of Hot Flushes. J Clin Endocr Metab. 2010;95(3):1199-206. doi: 10.1210/jc.2009-2262. PubMed PMID: WOS:000275197500027.

6. Casiglia E, Tikhonoff V, Mormino P, Piccoli A, Pessina AC. Is menopause an independent cardiovascular risk factor? Evidence from population-based studies. J Hypertens. 2002;20:S17-S22. PubMed PMID: WOS:000176186100005.

7. Choi SH, Lee SM, Kim Y, Choi NK, Cho YJ, Park BJ. Natural menopause and risk of stroke in elderly women. J Korean Med Sci. 2005;20(6):1053-8. Epub 2005/12/20. doi: 2005121053 [pii]

10.3346/jkms.2005.20.6.1053. PubMed PMID: 16361821; PubMed Central PMCID: PMC2779308.

8. Cui R, Iso H, Toyoshima H, Date C, Yamamoto A, Kikuchi S, et al. Relationships of age at menarche and menopause, and reproductive year with mortality from cardiovascular disease in Japanese postmenopausal women: The JACC study. J Epidemiol. 2006;16(5):177-84. doi: DOI 10.2188/jea.16.177. PubMed PMID: WOS:000240264600001.

9. Dallongeville J, Marecaux N, Isorez D, Zylbergberg G, Fruchart JC, Amouyel P. Multiple coronary heart disease risk factors are associated with menopause and influenced by substitutive hormonal therapy in a cohort of French women. Atherosclerosis. 1995;118(1):123-33. Epub 1995/11/01. doi: 0021-9150(95)05599-R [pii]. PubMed PMID: 8579622.

10. Gallicchio L, Miller SR, Zacur H, Flaws JA. Hot flashes and blood pressure in midlife women. Maturitas. 2010;65(1):69-74. Epub 2009/12/01. doi: S0378-5122(09)00394-6 [pii]

10.1016/j.maturitas.2009.10.013. PubMed PMID: 19945805; PubMed Central PMCID: PMC2815260.

11. Gast GC, Grobbee DE, Pop VJ, Keyzer JJ, Wijnands-van Gent CJ, Samsioe GN, et al. Menopausal complaints are associated with cardiovascular risk factors. Hypertension. 2008;51(6):1492-8. Epub 2008/04/09. doi: HYPERTENSIONAHA.107.106526 [pii]

10.1161/HYPERTENSIONAHA.107.106526. PubMed PMID: 18391100.

12. Gast GC, Samsioe GN, Grobbee DE, Nilsson PM, van der Schouw YT. Vasomotor symptoms, estradiol levels and cardiovascular risk profile in women. Maturitas. 2010;66(3):285-90. Epub 2010/04/20. doi: S0378-5122(10)00124-6 [pii]

10.1016/j.maturitas.2010.03.015. PubMed PMID: 20400247.

13. Gerber LM, Sievert LL, Warren K, Pickering TG, Schwartz JE. Hot flashes are associated with increased ambulatory systolic blood pressure. Menopause. 2007;14(2):308-15. Epub 2007/01/11. doi: 10.1097/01.gme.0000236938.74195.c6. PubMed PMID: 17213753.

14. Huang AJ, Grady D, Jacoby VL, Blackwell TL, Bauer DC, Sawaya GF. Persistent hot flushes in older postmenopausal women. Arch Intern Med. 2008;168(8):840-6. Epub 2008/04/30. doi: 168/8/840 [pii]

10.1001/archinte.168.8.840. PubMed PMID: 18443259.

15. Ingelsson E, Lundholm C, Johansson AL, Altman D. Hysterectomy and risk of cardiovascular disease: a population-based cohort study. Eur Heart J. 2011;32(6):745-50. Epub 2010/12/28. doi: ehq477 [pii]

10.1093/eurheartj/ehq477. PubMed PMID: 21186237.

16. Jacobsen BK, Heuch I, Kvale G. Age at natural menopause and stroke mortality - Cohort study with 3561 stroke deaths during 37-year follow-up. Stroke. 2004;35(7):1548-51. doi: 10.1161/01.STR.0000131746.49082.5c. PubMed PMID: WOS:000222257300004.

17. Kagitani H, Asou Y, Ishihara N, Hoshide S, Kario K. Hot flashes and blood pressure in middle-aged Japanese women. Am J Hypertens. 2014;27(4):503-7. Epub 2013/08/15. doi: hpt125 [pii]

10.1093/ajh/hpt125. PubMed PMID: 23942653.

18. Lee JS, Hayashi K, Mishra G, Yasui T, Kubota T, Mizunuma H. Independent association between age at natural menopause and hypercholesterolemia, hypertension, and diabetes mellitus: Japan nurses' health study. J Atheroscler Thromb. 2013;20(2):161-9. Epub 2012/10/20. doi: DN/JST.JSTAGE/jat/14746 [pii]. PubMed PMID: 23079582.

19. Lejskova M, Alusik S, Valenta Z, Adamkova S, Pitha J. Natural Postmenopause Is Associated With an Increase in Combined Cardiovascular Risk Factors. Physiol Res. 2012;61(6):587-96. PubMed PMID: WOS:000314138600004.

20. Lisabeth LD, Beiser AS, Brown DL, Murabito JM, Kelly-Hayes M, Wolf PA. Age at Natural Menopause and Risk of Ischemic Stroke The Framingham Heart Study. Stroke. 2009;40(4):1044-9. doi: 10.1161/Strokeaha.108.542993. PubMed PMID: WOS:000264709500005.

21. Lokkegaard E, Jovanovic Z, Heitmann BL, Keiding N, Ottesen B, Pedersen AT. The association between early menopause and risk of ischaemic heart disease: Influence of Hormone Therapy. Maturitas. 2006;53(2):226-33. doi: 10.1016/j.maturitas.2005.04.009. PubMed PMID: WOS:000234764000013.

22. Matthews KA, Schott LL, Bromberger J, Cyranowski J, Everson-Rose SA, Sowers MF. Associations between depressive symptoms and inflammatory/hemostatic markers in women during the menopausal transition. Psychosom Med. 2007;69(2):124-30. doi: 10.1097/01.psy.0000256574.30389.lb. PubMed PMID: WOS:000244804800002.

23. Matthews KA, Wing RR, Kuller LH, Meilahn EN, Plantinga P. Influence of the Perimenopause on Cardiovascular Risk-Factors and Symptoms of Middle-Aged Healthy Women. Archives of Internal Medicine. 1994;154(20):2349-55. doi: DOI 10.1001/archinte.154.20.2349. PubMed PMID: WOS:A1994PN18500009.

24. Mondul AM, Rodriguez C, Jacobs EJ, Calle EE. Age at natural menopause and cause-specific mortality. Am J Epidemiol. 2005;162(11):1089-97. doi: 10.1093/aje/kwi324. PubMed PMID: WOS:000233488900006.

25. Ossewaarde ME, Bots ML, Verbeek ALM, Peeters PHM, van der Graaf Y, Grobbee DE, et al. Age at menopause, cause-specific mortality and total life expectancy. Epidemiology. 2005;16(4):556-62. doi: 10.1097/01.ede.0000165392.35273.d4. PubMed PMID: WOS:000230068000020.

26. Parker WH, Broder MS, Chang E, Feskanich D, Farquhar C, Liu ZM, et al. Ovarian Conservation at the Time of Hysterectomy and Long-Term Health Outcomes in the Nurses' Health Study. Obstet Gynecol. 2009;113(5):1027-37. PubMed PMID: WOS:000265451700009.

27. Pines A. Vasomotor symptoms and cardiovascular disease risk. Climacteric. 2011;14(5):535-6. doi: 10.3109/13697137.2011.599058. PubMed PMID: WOS:000294827100004.

28. Raikkonen K, Matthews KA, Sutton-Tyrrell K, Kuller LH. Trait anger and the metabolic syndrome predict progression of carotid atherosclerosis in healthy middle-aged women. Psychosom Med. 2004;66(6):903-8. doi: 10.1097/01.psy.0000143638.31297.11. PubMed PMID: WOS:000225347900016.

29. Rivera CM, Grossardt BR, Rhodes DJ, Brown RD, Roger VL, Melton LJ, et al. Increased cardiovascular mortality after early bilateral oophorectomy. Menopause-the Journal of the North American Menopause Society. 2009;16(1):15-23. doi: 10.1097/gme.0b013e31818888f7. PubMed PMID: WOS:000262333100007.

30. Rocca WA, Grossardt BR, Miller VM, Shuster LT, Brown RD. Premature menopause or early menopause and risk of ischemic stroke. Menopause-the Journal of the North American Menopause Society. 2012;19(3):272-7. doi: 10.1097/gme.0b013e31822a9937. PubMed PMID: WOS:000300854000006.

31. Sassarini J, Fox H, Ferrell W, Sattar N, Lumsden MA. Vascular function and cardiovascular risk factors in women with severe flushing. Clin Endocrinol. 2011;74(1):97-103. doi: 10.1111/j.1365-2265.2010.03921.x. PubMed PMID: WOS:000285355500013.

32. Tehrani FR, Behboudi-Gandevani S, Ghanbarian A, Azizi F. Effect of menopause on cardiovascular disease and its risk factors: a 9-year follow-up study. Climacteric. 2014;17(2):164-72. doi: 10.3109/13697137.2013.828197. PubMed PMID: WOS:000333036600008.

33. Thurston RC, El Khoudary SR, Sutton-Tyrrell K, Crandall CJ, Gold E, Sternfeld B, et al. Are vasomotor symptoms associated with alterations in hemostatic and inflammatory markers? Findings from the Study of Women's Health Across the Nation. Menopause-the Journal of the North American Menopause Society. 2011;18(10):1044-51. doi: 10.1097/gme.0b013e31821f5d39. PubMed PMID: WOS:000295160300005.

34. Thurston RC, El Khoudary SR, Sutton-Tyrrell K, Crandall CJ, Gold EB, Sternfeld B, et al. Vasomotor Symptoms and Lipid Profiles in Women Transitioning Through Menopause. Obstet Gynecol. 2012;119(4):753-61. doi: 10.1097/AOG.0b013e31824a09ec. PubMed PMID: WOS:000301971400010.

35. Thurston RC, Kuller LH, Edmundowicz D, Matthews KA. History of hot flashes and aortic calcification among postmenopausal women. Menopause-the Journal of the North American Menopause Society. 2010;17(2):256-61. doi: 10.1097/gme.0b013e3181c1ad3d. PubMed PMID: WOS:000275485200010.

36. Thurston RC, Sutton-Tyrrell K, Everson-Rose SA, Hess R, Matthews KA. Hot flashes and subclinical cardiovascular disease - Findings from the Study of Women's Health Across the Nation Heart Study. Circulation. 2008;118(12):1234-40. doi: 10.1161/Circulationaha.108.776823. PubMed PMID: WOS:000259224800004.

37. Thurston RC, Sutton-Tyrrell K, Everson-Rose SA, Hess R, Powell LH, Matthews KA. Hot flashes and carotid intima media thickness among midlife women. Menopause-the Journal of the North American Menopause Society. 2011;18(4):352-8. doi: 10.1097/gme.0b013e3181fa27fd. PubMed PMID: WOS:000288781800005.

38. Tom SE, Cooper R, Wallace RB, Guralnik JM. Type and Timing of Menopause and Later Life Mortality Among Women in the Iowa Established Populations for the Epidemiological Study of the Elderly Cohort. J Womens Health. 2012;21(1):10-6. doi: 10.1089/jwh.2011.2745. PubMed PMID: WOS:000299134500003.

39. Tuomikoski P, Mikkola TS. Postmenopausal hormone therapy and coronary heart disease in early postmenopausal women. Annals of Medicine. 2014;46(1):1-7. doi: 10.3109/07853890.2013.854982. PubMed PMID: WOS:000329869800001.

40. Tuomikoski P, Mikkola TS, Hamalainen E, Tikkanen MJ, Turpeinen U, Ylikorkala O. Biochemical markers for cardiovascular disease in recently postmenopausal women with or without hot flashes. Menopause. 2010;17(1):145-51. Epub 2009/07/16. doi: 10.1097/gme.0b013e3181acefd5. PubMed PMID: 19602991.

41. Tuomikoski P, Mikkola TS, Tikkanen MJ, Ylikorkala O. Hot flushes and biochemical markers for cardiovascular disease: a randomized trial on hormone therapy. Climacteric. 2010;13(5):457-66. Epub 2010/05/07. doi: 10.3109/13697131003624656. PubMed PMID: 20443719.

42. Wolff EF, He YX, Black DM, Brinton EA, Budoff MJ, Cedars MI, et al. Self-reported menopausal symptoms, coronary artery calcification, and carotid intima-media thickness in recently menopausal women screened for the Kronos early estrogen prevention study (KEEPS). Fertil Steril. 2013;99(5):1385-91. doi: 10.1016/j.fertnstert.2012.11.053. PubMed PMID: WOS:000317393700044.
