# Supplementary figures and images for: Association of Vasomotor and Other Menopausal Symptoms with Risk of Cardiovascular Disease: A Systematic Review and Meta-Analysis
Source: PLoS One. 2016 Jun 17;11(6):e0157417. doi: 10.1371/journal.pone.0157417 (PMC4912069; doi:10.1371/journal.pone.0157417)

Basic model

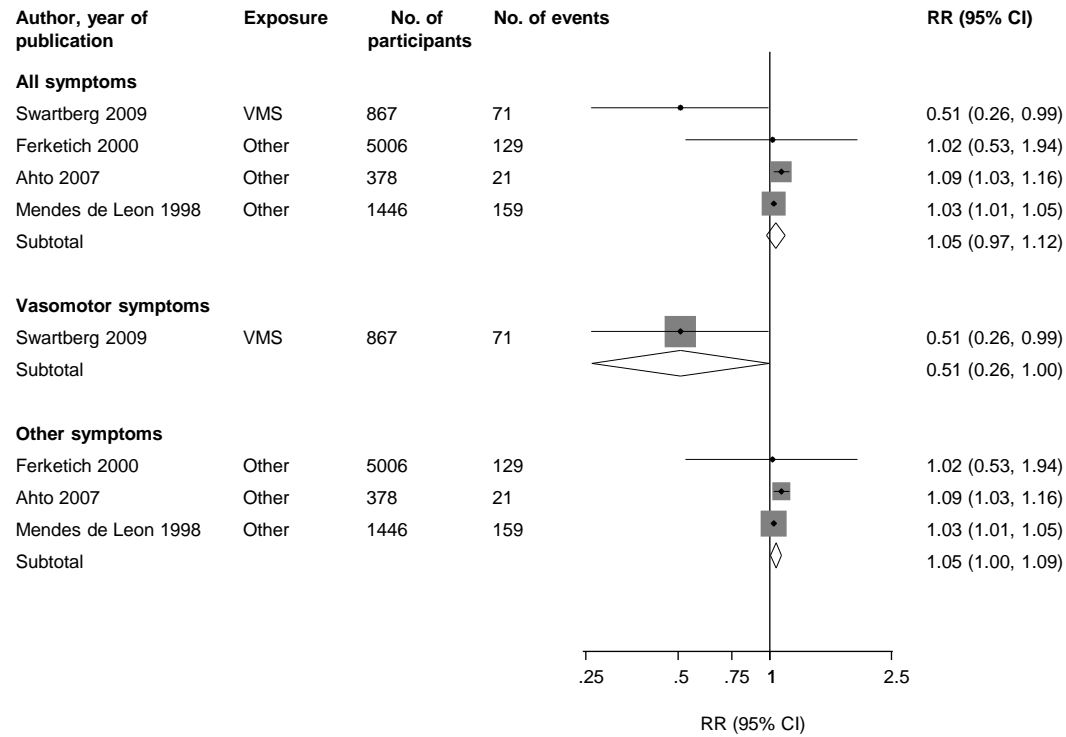

Fully-adjusted model

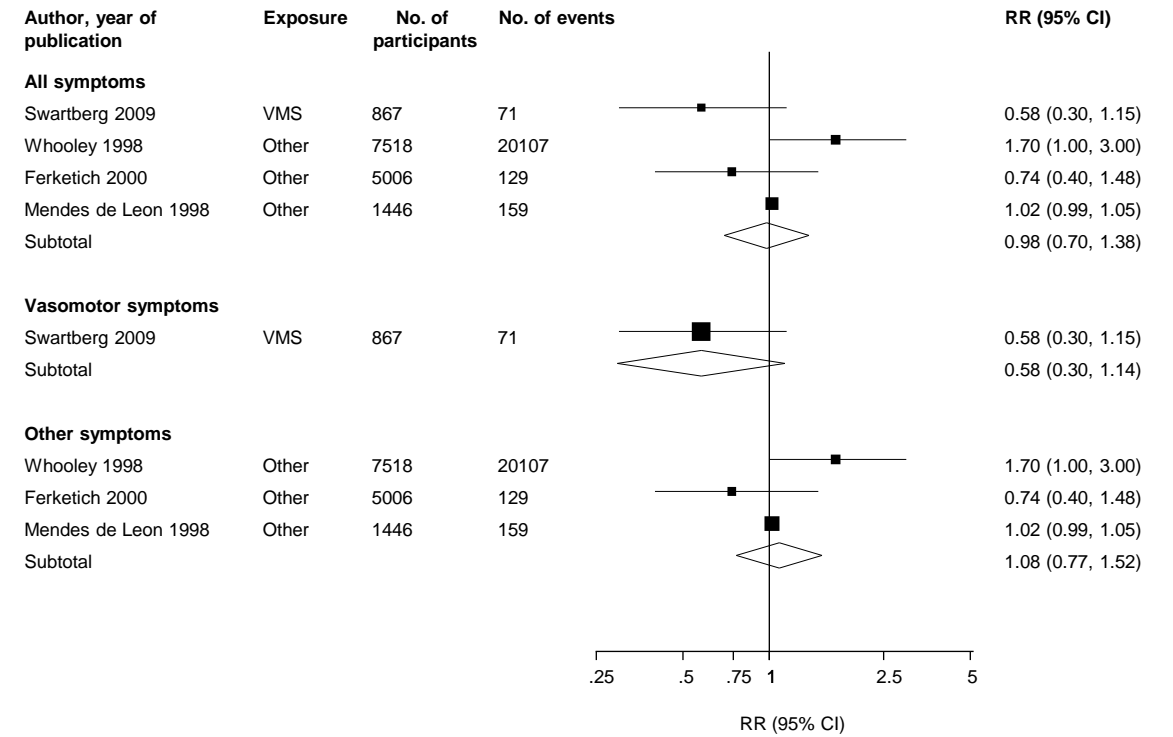

Supplement: S1 Fig — Basic model: adjusted for age and non-established cardiovascular risk factors; Fully-adjusted model, adjusted for established cardiovascular risk factors and potential mediators. VMS, vasomotor symptoms; vasomotor symptoms include hot flashes and /or night sweats; All, includes vasomotor and other menopausal symptoms; Other, includes menopausal symptoms such as depression, insomnia, and panic attacks. (PDF) [file pone.0157417.s005.pdf]

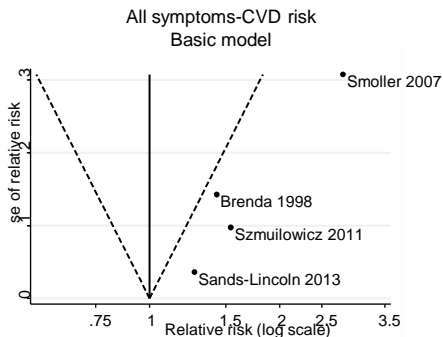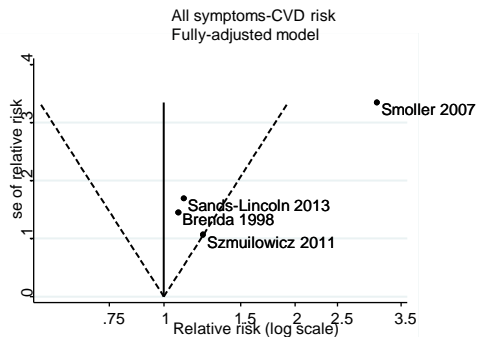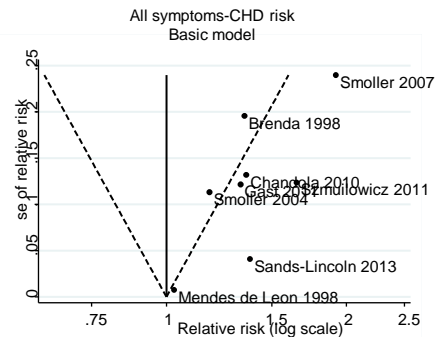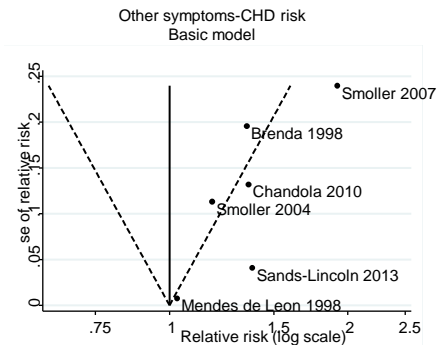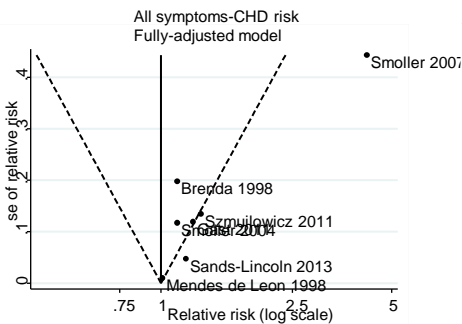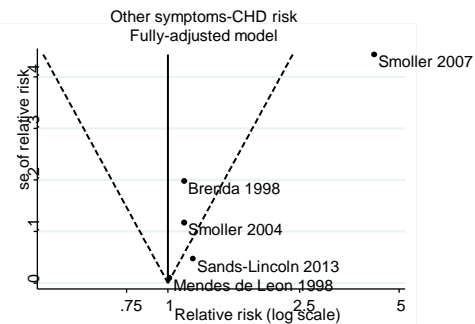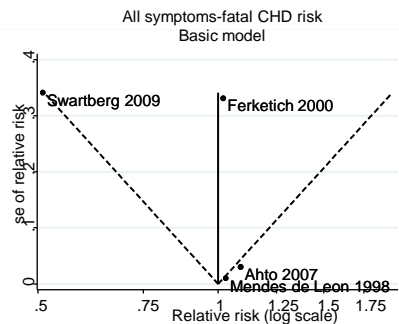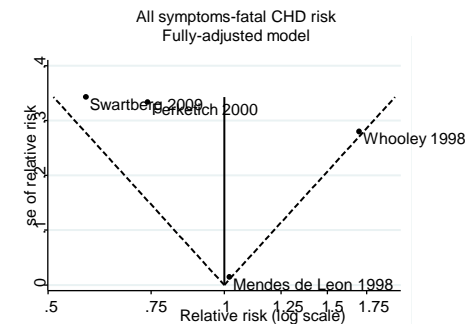

Supplement: S2 Fig — The dotted lines show 95% confidence intervals around the overall summary estimate calculated using a fixed effect model;; P-values for bias calculated using Egger’s test was 0.070 for all menopausal symptoms-CVD risk in basic adjusted analysis; 0.041 for all menopausal symptoms-CVD risk in fully- adjusted analysis; 0.016 for all menopausal symptoms-CHD risk in basic adjusted analysis; 0.083 for other menopausal symptoms-CHD risk in basic adjusted analysis; 0.014 for all menopausal symptoms-CHD risk in fully-adjusted analysis; 0.098 for other menopausal symptoms-CHD risk in fully-adjusted analysis; 0.599 for all menopausal symptoms-fatal CHD risk in basic adjusted analysis; and 0.824 for all menopausal symptoms-fatal CHD risk in fully-adjusted analysis respectively. (PDF) [file pone.0157417.s006.pdf]
