# Supplementary material for: Association of Vasomotor and Other Menopausal Symptoms with Risk of Cardiovascular Disease: A Systematic Review and Meta-Analysis
Source: PLoS One. 2016 Jun 17;11(6):e0157417. doi: 10.1371/journal.pone.0157417 (PMC4912069; doi:10.1371/journal.pone.0157417)
Supplement: S1 Table — (DOCX) [file pone.0157417.s007.docx]

| Lead Author, Publication Date | Exposure | Method used to assess the exposure |
| --- | --- | --- |
|  |  |  |
| Ahto, 2007 | Depression | Zung Self-Rating Depression Scale |
| Penninx, 1998; Mendes de Leon, 1998 | Depression | The Center for Epidemiologic Studies Depression Scale |
| Chandola, 2010 | Sleep disturbance | General Heath Questionnaire |
| Ferketich, 2000 | Depression | CES-D Scale- a Self-report depression scale |
| Gast, 2011 | Vasomotor Symptoms Presence in the previous week or during the preceding 3 months | Questionnaire |
| Sands-Lincoln, 2013 | Insomnia | WHI Insomnia Rating Scale (WHIIRS) |
| Smoller, 2007 | Panic Attacks in the previous 6 months | Questionnaire |
| Svartberg, 2009 | Vasomotor Symptoms | Self-administered questionnaire |
| Whooley, 1998 | Depression | The Geriatric Depression Scale short form- a self-report questionnaire |
| W-Smoller, 2004; Szmuilowicz 2011 | Late Vasomotor Symptoms (also early and persistent vasomotor symptoms) | Self-administered questionnaire |
